# Efficacy and Safety of Combined Topical Lidocaine and Tetracaine Cream for Facial Fractional Laser Resurfacing Compared With Its Reference Product in Chinese Adults: A Multicenter, Randomized, Double‐Blind Phase 3 Study

**DOI:** 10.1111/jocd.70358

**Published:** 2025-07-30

**Authors:** Wenxin Yu, Zhongling Luo, Yi Zhao, Zhiqi Hu, Aijun Zhang, Yong Li, Juan Tao, Xiaosong Chen, Haiying Wang, Liying Chen, Hanjie Zhu, Xiaoxi Lin

**Affiliations:** ^1^ Department of Laser and Aesthetic Medicine Shanghai Ninth People's Hospital, Shanghai Jiao Tong University School of Medicine Shanghai China; ^2^ Department of Dermatology Xiangya Hospital, Central South University Changsha China; ^3^ Department of Dermatology Beijing Tsinghua Changgung Hospital Beijing China; ^4^ Department of Plastic Surgery Nanfang Hospital, Southern Medical University Guangzhou China; ^5^ Department of Plastic Surgery Affiliated Hospital of Xuzhou Medical University Xuzhou China; ^6^ Department of Dermatology and Venereology West China Hospital, Sichuan University Chengdu China; ^7^ Department of Dermatology and Venereology Union Hospital Affiliated to Tongji Medical College, Huazhong University of Science and Technology Wuhan China; ^8^ Department of Plastic Surgery and Regenerative Medicine Fujian Medical University Union Hospital Fuzhou China; ^9^ Department of Dermatology and Venereology Peking University Shougang Hospital Beijing China; ^10^ Cutia Therapeutics Co., Ltd Wuxi China; ^11^ Department of Plastic and Reconstructive Surgery Shanghai Ninth People's Hospital, Shanghai Jiao Tong University School of Medicine Shanghai China

**Keywords:** efficacy, equivalence, fractional laser, lidocaine, safety, tetracaine

## Abstract

**Background:**

As a potentially effective anesthetic, CU‐30101 is comprised of 7% lidocaine and 7% tetracaine and is a generic drug of Pliaglis. This study aimed to investigate the equivalence between CU‐30101 and Pliaglis as topical anesthetics for fractional laser.

**Methods:**

This Phase 3 trial recruited Chinese adults scheduled for facial fractional laser resurfacing. Participants were randomized to receive opposite treatment sequences of CU‐30101 and Pliaglis on contralateral sides of the face prior to laser application. Visual Analog Scale (VAS), participant and investigator satisfaction ratings, local tolerability, and safety were assessed.

**Results:**

Two hundred eighty‐four participants completed the study, with mean VAS scores of 35.3 ± 24.51 for CU‐30101 and 37.3 ± 24.17 for Pliaglis. The 95% confidence interval of −3.78 to −0.13 for the VAS difference laid within the predefined equivalence margin of ±4.7, confirming equivalence in efficacy. The results in the per‐protocol set further corroborated the equivalence. Both participants and investigators expressed consistently high satisfaction with CU‐30101 and Pliaglis. Furthermore, both treatments were well tolerated and exhibited favorable safety profiles.

**Conclusion:**

This study demonstrated the equivalent efficacy of CU‐30101 to its reference product, Pliaglis, among Chinese adults undergoing facial fractional laser resurfacing. CU‐30101 showed commendable local tolerability and safety profiles, consistent with those of Pliaglis.

## 1 Introduction

Laser technology has significantly transformed the field of aesthetic medicine by providing a spectrum of treatments that ranges from non‐invasive to minimally invasive approaches [1, 2]. Fractional laser therapy, in particular, has become a distinguished modality. It utilizes localized photothermal effects to create microscopic thermal zones (MTZ) within the skin [3, 4]. The minimal size of MTZ injuries substantially reduces collateral damage to adjacent tissues [5], diminishes the risks associated with treatment, and accelerates recovery times [6]. Unlike traditional laser therapies, fractional laser technology offers wider clinical applicability. However, it is not without drawbacks, as it often involves varying degrees of pain and discomfort during treatment sessions [7]. Topical anesthesia has become an indispensable component of laser skin rejuvenation procedures due to its straightforward application and its efficacy in reducing procedural discomfort [8].

Lidocaine and tetracaine are widely recognized anesthetics that have proven highly effective in providing anesthesia and analgesia [9, 10, 11]. Pliaglis, a topical cream formulation containing 7% lidocaine and 7% tetracaine, has been available in the United States and European Union for several years [12, 13, 14]. Unlike other cream‐based anesthetic products, the formulation of Pliaglis allows for it to automatically form a film after exposure to the air, thus avoiding the need for additional occlusive dressing. Moreover, the film can be easily peeled off. Despite its proven efficacy, Pliaglis has not yet been introduced in China, where there is also a noticeable lack of approved lidocaine and tetracaine cream products. CU‐30101, a newly developed topical cream, contains the same active ingredients as Pliaglis. CU‐30101 has an identical concentration of 7% lidocaine (2.1 g) and 7% tetracaine (2.1 g) in a 30 g formulation. The excipients of CU‐30101 and Pliaglis are also identical. The film‐forming property of CU‐30101 can be achieved by its excipients, including polyvinyl alcohol and anhydrous dibasic calcium phosphate in a specific ratio. Thus, CU‐30101 exhibits a similar characteristic to Pliaglis in its ability to form a “peel‐off” film after exposure to the air and does not dry out within the specified usage time. CU‐30101 presents as a potential generic alternative and requires thorough investigation for providing effective analgesia during dermatologic procedures. Therefore, assessing the analgesic efficacy and safety profile of CU‐30101 in comparison to its reference product, Pliaglis, is an essential step forward in enhancing its clinical utility.

In this multicenter, randomized, double‐blind, phase 3 study, we aimed to rigorously evaluate whether CU‐30101 can offer equivalent efficacy and comparable safety with its reference product, Pliaglis.

## 2 Methods

### 2.1 Study Design and Participants

This study (NCT05793892) was a multicenter, randomized, double‐blind, active‐controlled, paired‐design phase 3 trial. It aimed to investigate the equivalent efficacy of CU‐30101 with its reference product and to evaluate its safety. Participants were enrolled across nine medical centers in China, from April 3, 2023, to August 25, 2023. Eligible participants were adults aged 18–65 years who were scheduled for fractional laser facial procedures due to reasons including acne scars, wrinkles, enlarged pores, and requesting overall skin texture improvement, and so forth. Those with facial skin issues or other medical conditions that could influence the study outcomes were excluded. More specific inclusion and exclusion criteria were available in the Supporting Information.

This study was conducted in adherence to the International Conference on Harmonization‐Good Clinical Practice (ICH‐GCP) guidelines and the ethical principles delineated in the Declaration of Helsinki. This study was approved by the ethics committee of Shanghai Ninth People's Hospital (Approval No. SH9H‐2022‐C71‐2) and each participating hospital, and informed consent was obtained from all participants.

### 2.2 Procedures

Randomization was performed using SAS EG 8.2 (SAS 9.4) with a block size of 10, employing a block randomization approach without stratification. The randomization sequence was generated by an independent statistician using SAS software, based on a pre‐set seed number, to create the final randomization list. The randomization sequence was managed by an interactive web response system (IWRS), ensuring a 1:1 allocation ratio between the two treatment sequence groups (Sequence A and Sequence B). The SAS program used for randomization (including the seed number) and the final randomization list are documented in the trial records, ensuring full reproducibility. To preserve blinding, drug application was performed by designated non‐blinded personnel. While uniform external packaging was used for both the test (CU‐30101) and reference (Pliaglis) products, the internal packaging differed (e.g., aluminum tube length and diameter of the tube openings). To prevent bias, non‐blinded drug administrators were responsible for applying and removing the drugs, while participants were blindfolded during the process. Non‐blinded personnel were strictly instructed not to discuss the color, texture, or packaging of the drugs with participants. After the drugs were removed from both cheeks, only then were blinded investigators permitted to interact with the subjects and conduct the fractional laser procedure, ensuring that both the investigators and participants remained fully blinded throughout the treatment process.

Considering the wide variety of lasers available and operator preferences, the type of fractional laser was not restricted in this study. The fractional lasers involved in the study included the 1565 nm fractional laser, CO_2_ fractional laser, etc. Treatment was symmetrically applied to the left and right sides of the face, with the drug applied at a thickness of approximately 1 mm and a tailored dose according to the size of the treatment area (1.3–1.5 g/10 cm^2^). Initially, the drugs were administered to the right side, followed by the left, with Sequence A receiving CU‐30101 on the right and Pliaglis on the left, and Sequence B receiving the opposite study drugs. Following a 30‐min application period, the drugs were removed in the same sequential order. Local tolerability assessments were performed by both investigators and participants after drug removal and prior to the fractional laser treatment on each side. The parameters for fractional laser treatment were determined by investigators according to the facial skin issues of the participants. The fractional laser treatment was applied to both sides with consistent parameters, commencing with the right side of the face and then proceeding to the left. Immediately following the completion of each side of the facial laser procedure, participants reported their pain using the Visual Analog Scale (VAS) for the corresponding side of the face, and satisfaction assessments were conducted by both participants and investigators. Safety was assessed throughout the treatment procedures. A follow‐up visit was scheduled on the third day post‐treatment.

### 2.3 Study Endpoints

The primary efficacy endpoint in this study was the pain assessment using the VAS, and comparing the pain levels in the CU‐30101‐treated area to the Pliaglis‐treated area on the facial regions subjected to fractional laser. The VAS scores were quantified using a 100‐mm scale, where 0 represented “no pain” and 100 signified the “most pain imaginable,” immediately following the completion of each side of facial laser procedure.

The secondary efficacy endpoints included the satisfaction assessments of both participants and investigators regarding the degree of anesthesia provided at both facial sides post‐fractional laser procedure, with specific questions provided in the Supporting Information.

Other secondary endpoints were local tolerability and safety, assessed by tolerability evaluation at the application site, records of adverse events (AEs), vital signs monitoring, physical examinations, laboratory tests, and 12‐lead electrocardiograms (ECGs). The evaluation of local tolerability at the application site at each facial side was performed by participants and investigators following the removal of the study drugs, prior to the laser treatment. This assessment was guided by the criteria set forth in the Chinese Expert Consensus on Adverse Reactions Evaluation of Skin Topical Medicine [15]. It involved grading both cutaneous manifestations and subjective symptoms associated with topical drug application. A 4‐point scale was utilized, ranging from 0 to 3 with increasing severity (Table S1). Adverse events (AEs) were assessed separately for facial AEs on the CU‐30101–treated and the Pliaglis‐treated sides, while non‐facial AEs were recorded individually rather than by two facial sides. Considering the potential confounders such as inflammatory reactions at the treatment site post‐fractional laser therapy, AEs were categorized as pre‐ and post‐fractional laser. The severity of AE (mild, moderate, severe) was determined by investigators based on the extent to which the AE impacted daily activities and the necessity for clinical intervention.

### 2.4 Statistical Analyses

The sample size was determined at 286 based on the assumption of equivalence between the CU‐30101 and Pliaglis treatment sides, with the anticipated mean of difference in VAS scores being 0. Utilizing the efficacy endpoint results from the reference product (NCT00110747) [16], the standard deviation (SD) for the difference was set at 22. The upper limit of the 95% confidence interval (CI) for the difference between the reference product and placebo was −9.53 (m1). In line with the principles outlined in the Chinese non‐inferiority trial design guidance for drug clinical trials, the fixed‐margin method was used to determine the non‐inferiority (equivalence) margin [17]. In alignment with these principles, the proportion (*f*) of retained treatment effect for Pliaglis was conservatively set at 0.5 (i.e., 50%), ensuring that the equivalence margin met regulatory standards while securing approval from China's regulatory authorities:

$$\mathrm{\Delta m}2=\left(1-f\right)\times m1$$

Accordingly, the equivalence margin was set at ±4.7. With a significance level (*α*) of 0.025 (for one‐sided tests) and a power of 85%, the required sample size was determined to be 256 using PASS16.0 software. Anticipating a drop‐out rate of no more than 10%, the total sample size was adjusted to 286 participants.

As the primary analysis dataset, full analysis set (FAS) included all randomized participants who received the study drugs and provided efficacy data for at least one side of the face. Per‐protocol set (PPS) comprised participants from the FAS who completed treatment and efficacy assessments and had no significant protocol deviations. Safety set (SS) included participants randomized and who had received the study drugs on at least one side of the face.

The equivalence of primary endpoints was assessed using a paired *t*‐test. Equivalence between the study drugs was established if the CI limits of the mean of difference in VAS scores between the paired facial sides (CU‐30101 relative to Pliaglis) fell within the predefined equivalence margin (±4.7). For intercurrent events (incomplete laser procedures on the left side of the face post‐medication), a composite variable strategy was employed for the primary analysis. If missing values persisted in the primary efficacy endpoints after applying the strategies corresponding to the intercurrent events, multiple imputation was then employed. Sensitivity analysis of the primary endpoints was performed using the original, unimputed VAS score data from the FAS. Supportive analysis of the primary endpoints was also conducted based on the PPS, with intercurrent events managed using the treatment policy strategy. No imputation was applied to the missing values of the secondary efficacy endpoints. Descriptive summaries of participant and investigator satisfaction assessments were derived from the FAS, and the consistency of satisfaction between the two drugs was analyzed using McNemar's test. The Bowker test was used to investigate the symmetry of the local tolerability assessment scores between two groups. A significance level of *p* < 0.05 was considered indicative of a statistical difference in VAS scores between the two sides. All analyses were performed using SAS EG 8.2 (SAS 9.4).

## 3 Results

### 3.1 Patient Characteristics and Treatment

**FIGURE 1:**
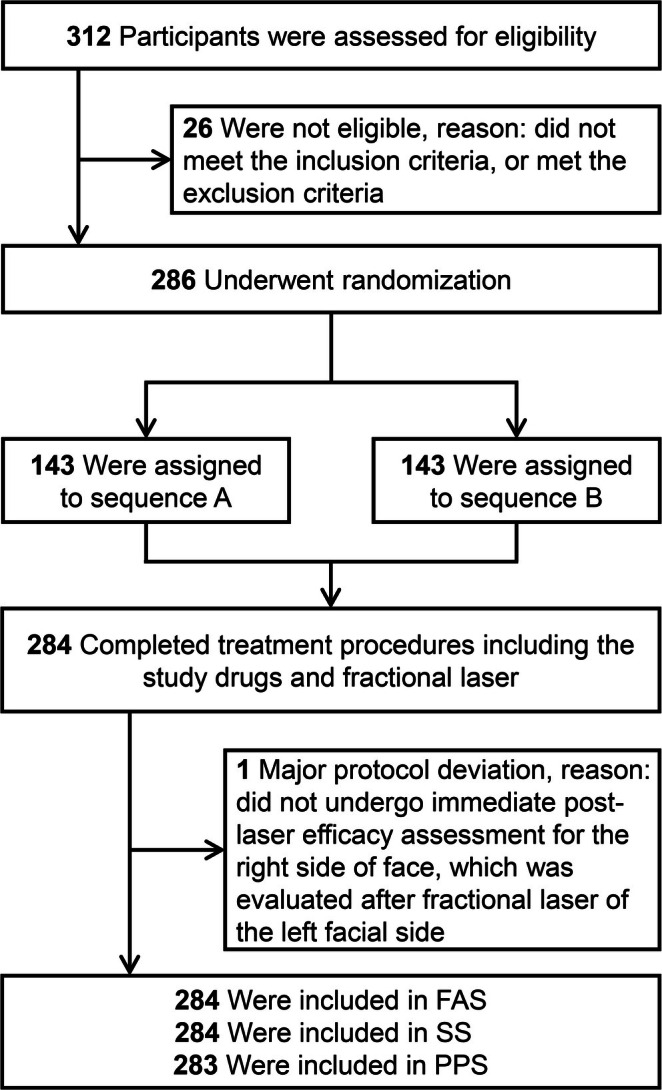
Flow diagram of the study. FAS, full analysis set; PPS, per‐protocol set; SS, safety set.

A total of 284 participants were finally included (Figure 1). All participants completed the treatment procedures involving the study drugs and fractional laser, and were included in both FAS and SS (*n* = 284). Notably, one participant failed to undergo immediate post‐laser efficacy assessment for the right side of the face, but was evaluated after fractional laser of the left facial side, and was thus excluded from the PPS (*n* = 283) due to this major protocol deviation. Ultimately, 283 participants completed the study, while one dropped out due to poor compliance and failure to attend a scheduled follow‐up visit.

As detailed in Table 1, the mean age of the 284 participants was 28.6 ± 6.14 years, with a predominance of females (220/284, 77.5%). Medical histories, including conditions such as acne and scars, as well as previous treatments, were consistently documented for both sides of the face. The administration of CU‐30101 and Pliaglis was uniform in terms of dosage and duration across both the left and right facial sides. The routine post‐laser therapies applied were comparable for both facial sides (Table S2).

**TABLE 1:** Demographics and baseline characteristics of participants (FAS).

Demographics and baseline characteristics	Value
Age, year (mean ± SD)	28.6 ± 6.14
Gender, male (*n*, %)	64 (22.5%)
Ethnicity, Han (*n*, %)	269 (94.7%)
BMI, kg/m^2^, (mean ± SD)	21.8 ± 2.65
History of allergies (*n*, %)	13 (4.6%)
Allergen (*n*, %)	
Lidocaine/tetracaine/amide or ester local anesthetics and adjuncts/*p*‐aminobenzoic acid	0
Others	13 (4.6%)
Smoking history (*n*, %)	9 (3.2%)

Abbreviations: BMI, body mass index; FAS, full analysis set; SD, standard deviation.

### 3.2 Primary Efficacy Endpoint

In the FAS, the mean VAS scores for the CU‐30101‐treated side and the Pliaglis‐treated side were 35.3 ± 24.51 and 37.3 ± 24.17, respectively (Table 2). The mean difference in VAS scores between the paired facial sides (CU‐30101 relative to Pliaglis) was −2.0 ± 15.63 (Table 2). A paired *t*‐test revealed that the 95% CI of −3.78 to −0.13 fell within the predefined equivalence margin, with a *p* value of 0.036, thus demonstrating equivalent efficacy between CU‐30101 and Pliaglis (Table 2).

**TABLE 2:** Summary of primary efficacy endpoint results (FAS).

VAS assessment	CU‐30101 side	Pliaglis side	Difference between the paired facial sides
	(*n* = 284)	(*n* = 284)	(CU‐30101 relative to Pliaglis)
*n*	284	284	284
Mean	35.3	37.3	−2.0
SD	24.51	24.17	15.63
Median	31.0	33.0	−1.0
Q1, Q3	15.0, 51.5	18.0, 53.0	−10.0, 7.0
Min, Max	0, 100	0, 100	−60, 63
95% CI for mean of difference			−3.78, −0.13
*p*			0.036

Abbreviations: CI, confidence interval; FAS, full analysis set; SD, standard deviation; VAS, Visual Analog Scale.

These findings were consistently supported in the PPS, where the mean VAS scores for CU‐30101 and Pliaglis were 35.2 ± 24.50 and 37.1 ± 24.12, respectively. The mean of difference in VAS scores between the paired facial sides (CU‐30101 relative to Pliaglis) was −1.9 ± 15.65, with a 95% CI of −3.75 to −0.09, and a *p* value of 0.040, further reinforcing the equivalent efficacy of CU‐30101 compared to Pliaglis.

Notably, throughout the study, no participants experienced incomplete fractional laser procedures on the left facial side due to AEs, precluding the necessity for intercurrent event handling and supportive analysis. Moreover, there were no missing VAS scores, and consequently, no imputation and sensitivity analysis were required.

### 3.3 Secondary Efficacy Endpoints

Within the FAS, all participants and investigators completed the secondary efficacy assessments. As detailed in Table 3, participants reported comparable levels of satisfaction with both study drugs. The McNemar test for both queried aspects yielded *p* values exceeding 0.05 (0.418 and 0.060), indicating consistency in the satisfaction ratings for the study drugs. Similarly, investigators demonstrated congruent satisfaction levels with both study drugs, with the *p* value of the McNemar test exceeding 0.05 (*p* = 0.210). The majority of participants and investigators considered that both study drugs provided sufficient anesthesia (Table 3).

**TABLE 3:** Secondary efficacy endpoints assessment (FAS).

Secondary efficacy assessments	Pliaglis side	CU‐30101 side			*p* for McNemar test
		Yes	No	Total	
Question for participants: “Did the study drug adequately relieved your pain?”					0.418
	Yes (*n*, %)	208 (73.2%)	16 (5.6%)	224 (78.9%)	
	No (*n*, %)	22 (7.7%)	38 (13.4%)	60 (21.1%)	
	Total (*n*, %)	230 (81.0%)	54 (19.0%)	284 (100%)	
Question for participants: “Will you consider using the study drug for topical anesthesia again?”					0.060
	Yes (*n*, %)	208 (73.2%)	17 (6.0%)	225 (79.2%)	
	No (*n*, %)	31 (10.9%)	28 (9.9%)	59 (20.8%)	
	Total (*n*, %)	239 (84.2%)	45 (15.8%)	284 (100%)	
Question for investigators: “Did the study drug provided sufficient anesthesia for the fractional laser procedure?”					0.210
	Yes (*n*, %)	240 (84.5%)	8 (2.8%)	248 (87.3%)	
	No (*n*, %)	15 (5.3%)	21 (7.4%)	36 (12.7%)	
	Total (*n*, %)	255 (89.8%)	29 (10.2%)	284 (100%)	

Abbreviation: FAS, full analysis set.

### 3.4 Local Tolerability and Safety

In the SS, local tolerability assessments for CU‐30101 and Pliaglis are summarized in Table S3. The Bowker test showed a *p* value of 0.754 for cutaneous manifestation scores, indicating no statistically significant difference between CU‐30101 and Pliaglis‐treated facial sides. Furthermore, the subjective symptom scores also showed no significant difference (*p* = 0.692).

Before the fractional laser treatments, the incidence rates of facial AEs were closely comparable for both CU‐30101 and Pliaglis sides, as detailed in Table 4, with rates of 44% and 42.6%, respectively. All reported facial AEs were considered related to study drugs. The majority of these AEs were mild, occurring in 37.0% of participants on the CU‐30101 side and 35.6% on the Pliaglis side, while a minority were classified as moderate, occurring in 7.0% of participants for both sides. There were no severe AEs, SAEs, death, nor AEs leading to withdrawal from the trial. All facial AEs resolved without sequelae. The facial AE profiles were similar between CU‐30101 and Pliaglis‐treated facial sides.

**TABLE 4:** Summary of facial adverse events (SS).

	Facial AEs before fractional laser		Facial AEs after fractional laser	
	CU‐30101	Pliaglis	CU‐30101	Pliaglis
	(*N* = 284)	(*N* = 284)	(*N* = 284)	(*N* = 284)
Any AE	125 (44.0%)	121 (42.6%)	17 (6.0%)	16 (5.6%)
Severity				
Mild	105 (37.0%)	101 (35.6%)	7 (2.5%)	7 (2.5%)
Moderate	20 (7.0%)	20 (7.0%)	10 (3.5%)	9 (3.2%)
Severe	0	0	0	0
AEs related with study drugs	125 (44.0%)	121 (42.6%)	4 (1.4%)	3 (1.1%)
SAE	0	0	0	0
AEs leading to hospitalization or prolonged hospital stay	0	0	0	0
AEs leading to study withdrawal	0	0	0	0
AEs leading to death	0	0	0	0
AEs with incidence > 1% on CU‐30101 or Pliaglis side before fractional laser				
Application site erythema	113 (39.8%)	112 (39.4%)	0	0
Study drugs‐related	113 (39.8%)	112 (39.4%)	0	0
Application site swelling	55 (19.4%)	58 (20.4%)	0	1 (0.4%)
Study drugs‐related	55 (19.4%)	58 (20.4%)	0	1 (0.4%)
Application site edema	14 (4.9%)	13 (4.6%)	0	0
Study drugs‐related	14 (4.9%)	13 (4.6%)	0	0
Application site pain	14 (4.9%)	7 (2.5%)	1 (0.4%)	1 (0.4%)
Study drugs‐related	14 (4.9%)	7 (2.5%)	0	0
Application site itching	2 (0.7%)	3 (1.1%)	2 (0.7%)	1 (0.4%)
Study drugs‐related	2 (0.7%)	3 (1.1%)	2 (0.7%)	1 (0.4%)
Burning sensation	4 (1.4%)	4 (1.4%)	0	0
Study drugs‐related	4 (1.4%)	4 (1.4%)	0	0
AEs with incidence > 1% on CU‐30101 or Pliaglis side after fractional laser				
Blister	1 (0.4%)	1 (0.4%)	4 (1.4%)	4 (1.4%)
Study drugs‐related	1 (0.4%)	1 (0.4%)	0	0

Abbreviations: AE, adverse event; SAE, serious AE; SS, safety set.

Following the fractional laser treatments, as depicted in Table 4, rates of facial AEs related to the study drugs were minimal, at 1.4% for CU‐30101 and 1.1% for Pliaglis. Except for one participant with a facial AE unrelated to the study drugs that did not resolve, all other cases had recovered. Additionally, 39 participants (13.7%) experienced non‐facial AEs after fractional laser treatments. These non‐facial AEs were classified as mild in the majority of cases, and there were only two cases with moderate AEs. Only one non‐facial AE, which was electrocardiogram ST‐segment depression, was determined by the investigator to be possibly related to both study drugs. The severity of this AE was mild.

## 4 Discussion

This randomized, double‐blind, positive‐controlled, paired‐design phase 3 equivalence trial has demonstrated the equivalent efficacy of CU‐30101 to its reference product, Pliaglis. Moreover, it has established favorable local tolerability and safety profiles for both drugs. The notable satisfaction among participants and investigators regarding the use of CU‐30101 in the context of fractional laser anesthesia further highlights its promising role in facial laser procedures. This not only broadens the potential anesthesia options available for such procedures but also suggests applications in other superficial dermatological procedures. These findings provide a robust foundation for further research and practical application of CU‐30101, aiming to enhance patient care and therapeutic outcomes in facial aesthetic treatments.

The primary efficacy outcome of this study confirms the equivalence between CU‐30101 and Pliaglis, with the 95% CI of the mean of VAS difference between the paired facial sides within the predefined equivalence margin. The analysis based on the PPS further substantiates this finding. The equivalence of the two treatments primarily hinges on various aspects of the study's design. Stringent enrollment criteria were employed to avoid potential confounders in assessing facial pain. The sample size was carefully determined based on rigorous pre‐study calculations, and strict adherence to the protocol was maintained, with only one instance of significant deviation noted, thereby ensuring sufficient statistical power to support the study's conclusions. Furthermore, the study's flexibility in not limiting the types of laser devices (including both ablative and non‐ablative fractional laser) or specific treatment parameters allowed clinicians to adapt treatment plans to the individual needs of participants. This approach enhances the applicability of the study findings to real‐world clinical settings. The selection of VAS scores as the primary endpoint for this study is well justified. VAS scores are widely used in clinical research [18, 19, 20], particularly in studies assessing the efficacy of anesthetics, due to their established reliability in measuring pain levels. Additionally, previous studies [21] assessing the reference product, Pliaglis, have consistently employed a 100 mm VAS scale to evaluate its efficacy in various laser therapies, further supporting the validity of VAS scores as a robust study endpoint. A recent study [22] on the treatment of post‐epicanthoplasty scarring in the medial canthal area with non‐ablative fractional laser reported a VAS score showing superior satisfaction in the treated group. VAS score has been used across different treatment contexts, for instance, in evaluating pain following fractional laser treatment of non‐facial areas with topical anesthesia of articaine/epinephrine solution [23]. In this study, the observed VAS score for CU‐30101 in facial fractional laser treatments reinforced its efficacy as an anesthetic and analgesic agent.

The primary objective of this study was to demonstrate the equivalence of CU‐30101 and Pliaglis in providing topical anesthesia for fractional laser resurfacing. The equivalence margin (±4.7 VAS points) was determined using statistical inference, as no universally accepted minimal clinically important difference (MCID) exists for facial pain in dermatological procedures. In the absence of a defined MCID for facial VAS scores, relevant literature suggests that for postoperative pain, a 30% reduction in VAS scores or an absolute reduction of 2–3 points (on a 10‐point scale, equivalent to about 12–15 points on a 100‐point scale) is often considered clinically meaningful [24, 25]. A recent study [26] identified an MCID of 12 points for facial pain, which significantly exceeds the observed VAS difference in this study (−2.0 points, 95% CI: −3.78 to −0.13). Additionally, previous VAS‐based assessments in facial surgery support that small VAS differences (< 5 points on a 100‐point scale) are unlikely to be clinically significant. This indicates that the observed VAS difference between CU‐30101 and Pliaglis is unlikely to be clinically meaningful, further supporting the equivalence.

The tolerability of CU‐30101 was favorable, paralleling that of Pliaglis, with the majority of cutaneous manifestations and subjective symptoms classified as mild to moderate in severity. The safety profile of CU‐30101 was consistent with that of Pliaglis. Overall, CU‐30101 exhibited favorable local tolerability and safety. Consistent with the prescribing information for Pliaglis and corroborated by real‐world data [13, 27], the facial AEs associated with CU‐30101 observed in this study were consistent with the established safety profile of Pliaglis. No new safety signals have arisen during the study. The favorable safety profile of CU‐30101 lays a strong foundation for its broader clinical application.

In the context of superficial dermatological procedures, such as fractional laser therapy, the demand for effective and safe anesthesia is paramount [28]. Although Pliaglis has been available in the United States and Europe for several years, its absence in the Chinese market highlights the need for alternative anesthetic options. Currently, the anesthetics predominantly used in facial laser procedures in China consist mainly of compounded lidocaine‐prilocaine creams [29, 30]. The introduction of CU‐30101, containing 7% lidocaine and 7% tetracaine, broadens the range of potential options available. This study has successfully demonstrated the equivalence of CU‐30101 to Pliaglis in terms of providing effective analgesia during fractional laser therapy, while also showcasing its commendable safety profile. Further investigation into its varied clinical applications is highly encouraged in diverse clinical settings.

A limitation of this study is that AEs occurring after laser treatment were assessed in relation to the study drug, without specific differentiation from procedure‐related effects. As the primary objective was to evaluate the safety of CU‐30101, this study did not determine whether post‐treatment AEs were attributable to the laser procedure itself. Additionally, the safety follow‐up period was limited to 3 days, mirroring the design of the original Pliaglis registration trial, which conducted 72‐h post‐treatment follow‐up via telephone. Given the extensive safety data available for Pliaglis, where reported AEs (e.g., erythema, skin discoloration, edema) were generally mild and self‐limiting, the 3‐day follow‐up period was deemed sufficient for assessing short‐term drug‐related safety. Moreover, since both facial sides received an active drug without a blank control, and laser treatment itself can cause transient skin irritation and barrier disruption, an extended safety follow‐up period might not have provided additional meaningful differentiation between drug‐ and laser‐induced effects. Future studies may consider longer follow‐up periods or dermatological assessments (e.g., barrier function recovery, prolonged erythema evaluation) to further characterize the long‐term safety profile of CU‐30101. Furthermore, while laser parameters (e.g., ablative vs. non‐ablative devices, energy settings) were identical within each subject, they were not standardized across centers, potentially introducing variability in pain perception between participants. Although the paired design minimized intra‐individual confounding, the lack of laser stratification may limit the generalizability of findings to specific laser modalities. Future studies should consider pre‐specifying laser treatment parameters or conducting subgroup analyses to validate equivalence across different laser technologies. A limitation of this study is the predominance of female participants, which may limit the generalizability of the findings to less‐represented gender groups, particularly men. While the self‐controlled design minimizes the impact of inter‐individual pain perception differences, future studies with a more balanced gender distribution are needed to confirm the equivalence of CU‐30101 and Pliaglis across both sexes. However, this limitation does not affect the study's primary conclusion regarding the equivalence of the two treatments.

In conclusion, this study demonstrates the equivalent efficacy of CU‐30101 to its reference product, Pliaglis, in the context of facial fractional laser treatments. Both investigators and participants reported high levels of satisfaction with the analgesic effects of CU‐30101. Furthermore, CU‐30101 demonstrated favorable local tolerability and a consistent safety profile with Pliaglis, without the emergence of new safety signals. The clinical equivalence of CU‐30101 to Pliaglis highlights its potential for further application in broader clinical settings.

## Author Contributions

**Wenxin Yu:** investigation, writing – review and editing. **Zhongling Luo, Yi Zhao, Zhiqi Hu, Aijun Zhang, Yong Li, Juan Tao, Xiaosong Chen, Haiying Wang:** investigation. **Liying Chen:** writing – review and editing. **Hanjie Zhu:** writing – review and editing, formal analysis. **Xiaoxi Lin:** investigation, writing – review and editing.

## Conflicts of Interest

Liying Chen and Hanjie Zhu are employed by Cutia Therapeutics Co. Ltd. Other authors declared no conflicts of interest.

## Supporting information


**Table S1.** Scores of cutaneous manifestations and subjective symptoms.
**Table S2.** Concurrent facial therapies at the CU‐30101 side and Pliaglis side (FAS).
**Table S3.** Summary of local tolerability assessment (SS).

## Data Availability

The data that supports the findings of this study are included in this published article and Supporting Information.
