# Supplementary material for: Efficacy and Safety of Combined Topical Lidocaine and Tetracaine Cream for Facial Fractional Laser Resurfacing Compared With Its Reference Product in Chinese Adults: A Multicenter, Randomized, Double‐Blind Phase 3 Study
Source: J Cosmet Dermatol. 2025 Jul 30;24(8):e70358. doi: 10.1111/jocd.70358 (PMC12308782; doi:10.1111/jocd.70358)
Supplement: Supplementary file 1 — Table S1. Scores of cutaneous manifestations and subjective symptoms. Table S2. Concurrent facial therapies at the CU‐30101 side and Pliaglis side (FAS). Table S3. Summary of local tolerability assessment (SS). [file JOCD-24-e70358-s001.docx]

**Supplementary Methods**

**Inclusion and exclusion criteria**

The inclusion criteria were: 1) Chinese adults aged 18 to 65 years; 2) Body mass index (BMI) ranging from 18.5 to 28.0 kg/m²; 3) Candidates scheduled for fractional laser facial procedures; 4) Comprehensive understanding of the study protocols and provision of informed consent.

The key exclusion criteria: 1) Presence of facial skin conditions that could influence the study outcomes, such as abrasions, peeling, tattoos, scars, atopic dermatitis, urticaria, or other relevant dermatological issues; 2) Medical conditions that could affect facial pain perception, including sensory disturbances, hyperalgesia, migraines, herpes zoster, or trigeminal neuralgia; 3) Use of any analgesics within 24 hours prior to the screening process; 4) Consistent use of analgesics in the past six months or a history of substance abuse; 5) History of alcohol abuse, regular alcohol consumption within the six months prior to screening, or a positive alcohol breath test at the time of screening; 6) Any other conditions identified by the investigators as unsuitable for participation in the study.

**Study drugs**

CU-30101 is composed of a 30g formulation containing 2.1g of lidocaine and 2.1g of tetracaine, and produced by Cutia Therapeutics (Wuxi) Co., Ltd with batch number of 21102701. The reference product, Pliaglis®, is similarly formulated, consisting of 30g formulation containing 2.1g of lidocaine and 2.1g of tetracaine, and manufactured by Difa Cooper S.p.A with batch numbers of S263 and S351A.

**Secondary efficacy endpoints: satisfaction assessments**

Participants responded to two binary questions: 1) "Did the study drug adequately relieved your pain?" 2) "Will you consider using the study drug for topical anaesthesia again?" Investigators answered a binary question: "Did the study drug provided sufficient anaesthesia for the fractional laser procedure?"

**Supplementary Tables**

**Table S1. Scores of cutaneous manifestations and subjective symptoms.**

| **Assessment** | **Score** | **Grading criteria** |
| --- | --- | --- |
| Cutaneous manifestation | | |
|  | 0 | None |
|  | 1 | Mild: Faint erythema without edema (lesions not palpable) and papules. |
|  | 2 | Moderate: Clear erythema with edema (lesions palpable) and papules. |
|  | 3 | Severe: Presence of vesicles, bullae, exudation or pustules, erosion, exudation or ulceration, or plaques, and thickening. |
| Subjective symptom (itchiness, pain or burning sensation) | | |
|  | 0 | None |
|  | 1 | Mild: Symptoms do not interfere with daily activities or sleep. |
|  | 2 | Moderate: Symptoms interfere with daily activities but not with sleep. |
|  | 3 | Severe: Symptoms interfere with sleep. |

**Table S2. Concurrent facial therapies at the CU-30101 side and Pliaglis® side (FAS)**

| **Concurrent facial therapies** | **CU-30101 side**  **(n=284)** | **Pliaglis® side**  **(n=284)** |
| --- | --- | --- |
| Wet compress therapy (n, %) | 251 (88.4%) | 251 (88.4%) |
| Cooling therapy (n, %) | 87 (30.6%) | 87 (30.6%) |
| Antibiotics treatment (n, %) | 11 (3.9%) | 11 (3.9%) |
| Light therapy (n, %) | 2 (0.7%) | 2 (0.7%) |

FAS: full analysis set.

**Table S3. Summary of local tolerability assessment (SS)**

| Local tolerability assessments | Pliaglis® side | CU-30101 side | | | | | P for Bowker test |
| --- | --- | --- | --- | --- | --- | --- | --- |
|  |  | 0 | 1 | 2 | 3 | Total |  |
| Cutaneous manifestation score |  |  |  |  |  |  | 0.754 |
|  | 0 (n, %) | 19 (6.7%) | 1 (0.4%) | 0 | 0 | 20 (7.0%) |  |
|  | 1 (n, %) | 1 (0.4%) | 181 (63.7%) | 3 (1.1%) | 0 | 185 (65.1%) |  |
|  | 2 (n, %) | 0 | 6 (2.1%) | 72 (25.4%) | 0 | 78 (27.5%) |  |
|  | 3 (n, %) | 0 | 0 | 0 | 1 (0.4%) | 1 (0.4%) |  |
|  | Total (n, %) | 20 (7.0%) | 188 (66.2%) | 75 (26.4%) | 1 (0.4%) | 284 (100%) |  |
| Subjective symptom score |  |  |  |  |  |  | 0.692 |
|  | 0 (n, %) | 101 (35.6%) | 5 (1.8%) | 0 | 0 | 106 (37.3%) |  |
|  | 1 (n, %) | 4 (1.4%) | 163 (57.4%) | 3 (1.1%) | 0 | 170 (59.9%) |  |
|  | 2 (n, %) | 0 | 1 (0.4%) | 7 (2.5%) | 0 | 8 (2.8%) |  |
|  | 3 (n, %) | 0 | 0 | 0 | 0 | 0 |  |
|  | Total (n, %) | 105 (37.0%) | 169 (59.5%) | 10 (3.5%) | 0 | 284 (100%) |  |

SS: safety set.
